# High intensity exercise before sleep boosts memory encoding the next morning

**DOI:** 10.1038/s41598-025-07880-z

**Published:** 2025-07-01

**Authors:** Daniela Ramirez Butavand, Juliane Nagel, Gordon B. Feld, Simon Steib

**Affiliations:** 1 Human Movement, Training and Active Aging Department, Institute of Sports and Sports Science, Heidelberg University, Heidelberg, Germany; 2 Clinical Psychology, Central Institute of Mental Health, Medical Faculty Mannheim, Heidelberg University, Mannheim, Germany; 3 Addiction Behavior and Addiction Medicine, Central Institute of Mental Health, Medical Faculty Mannheim, Heidelberg University, Mannheim, Germany; 4 Psychiatry and Psychotherapy, Central Institute of Mental Health, Medical Faculty Mannheim, Heidelberg University, Mannheim, Germany; 5 Psychological Institute, Heidelberg University, Heidelberg, Germany; 6 German Center for Mental Health (DZPG), Mannheim, Germany; 7 Network Aging Research, Heidelberg University, Heidelberg, Germany

**Keywords:** High-intensity interval training, Memory encoding, Sleep, Human behaviour, Learning and memory

## Abstract

The importance of sleep for memory consolidation has been extensively studied, but its role for memory encoding remains less well characterized. At the molecular and cellular level, the renormalization of synaptic weights during sleep has received substantial support, which is thought to free capacity to encode new information at the behavioral level. However, at the systems level and behaviorally, support for this process playing a major role for memory function remains scarce. In the current study, we investigated the utility of moderate- and high-intensity evening exercise as a low-cost low-tech intervention to modulate sleep and its influence on subsequent encoding in the morning. Our findings indicate that high-intensity interval training (HIIT) improved post-sleep memory performance with effects lasting up to 24 h after initial encoding. In addition, we show that especially the early parts of the encoding task were affected by the HIIT intervention. Intriguingly, participants with lower encoding abilities seemed to benefit more from the HIIT intervention suggesting it not only as a tool for basic research but also as a candidate for applications to boost memory performance in mental disorders or in the elderly. These results provide first evidence that acute exercise can affect learning processes even hours after it occurs.

**Supplementary Information:**

The online version contains supplementary material available at 10.1038/s41598-025-07880-z.

## Introduction

Sleep plays an important role for efficient memory processing^1–3^. During sleep, memories are strengthened and transformed via active systems consolidation, which relies on the repeated reactivation of memory traces that were encoded during prior wakefulness^4^. In addition, according to the synaptic homeostasis theory, the brain’s encoding capacity is replenished during sleep^5,6^. Taken together, it is suggested that sleep sorts out irrelevant information to enable efficient new learning and preserves relevant information for the long-term^7^. Identifying non-invasive interventions that can be used to manipulate the underlying neuronal processes and that enable cost-effective low-tech applications to improve human health and cognition is an important goal of sleep and memory research^8^. At the same time, these simple solutions allow more robust research on the behavioral effects of synaptic homeostasis since they allow larger sample sizes^9^. Utilizing exercise to enhance sleep and the associated cognitive processes is an ideal candidate for such an intervention^10^.

Behavioral interventions can improve memory, e.g., exposure to stress or novelty in natural settings^11,12^, as well as engaging in physical exercise^13–16^. Meta-analyses have revealed that acute exercise, defined as a single bout of physical activity, when administered in close temporal proximity to encoding, has a moderate to large effect on episodic and motor memory^17,18^. Notably, high-intensity interval training (HIIT), i.e., repeated short bouts of high-intensity exercise interspersed with recovery periods at low intensity, that combines high efficacy and low time investment, has been shown to improve memory encoding and consolidation^19–21^.

At the same time, it has been suggested that exercise can modify sleep, although here the evidence is less robust. Nonetheless, meta-analyses concluded that acute exercise affects objective measures of sleep quality and architecture, as well as neurophysiological sleep characteristics^22–25^. The most consistent finding is that acute exercise reduces REM sleep and increases non-REM sleep stage 2 (N2) and slow-wave sleep (SWS). Individual studies have also shown that vigorous exercise increases slow wave activity (SWA) and fast spindle activity^26–28^. Given sleep’s role in memory processing, it is highly plausible that exercise exerts its effects on memory via sleep enhancements. This novel hypothesis is supported by three recent studies^29–31^. Although exercise has been shown to enhance sleep-dependent memory consolidation, it has not yet been investigated whether exercise-induced modifications of sleep also enhance memory encoding the following day via the synaptic homeostasis mechanism.

A vast amount of molecular and cellular studies support the synaptic homeostasis theory^32–39^. In humans, the theory is supported by a few studies showing improved memory encoding after sleep versus wakefulness^40,41^ or sleep deprivation^42^, accompanied by increased activity in the hippocampus^41,42^. Moreover, two studies suggested that SWS, and especially the sleep slow oscillation, might be crucial for this effect of sleep in restoring encoding capacity. Antonenko and colleagues^43^ employed transcranial slow oscillation stimulation (tSOS) during a nap, resulting in enhanced SWA and improved encoding of several episodic memory tasks (object recognition, word pairs, and word lists). Conversely, Van Der Werf and colleagues^44^ attenuated SWA by acoustic perturbations during nighttime sleep in elderly participants. This manipulation led to worse post-sleep encoding preceding an image recognition task, accompanied by reduced hippocampal activation during encoding of later recalled items. Both of these interventions require a relatively sophisticated technical setup. Utilizing exercise as a simple intervention to enhance sleep’s restorative effect therefore represents an opportunity to study the basics of synaptic homeostasis theory at the systems level and ultimately develop novel cost-effective interventions targeting this mechanism.

In the present study, we examined the impact of two distinct evening exercise protocols on subsequent nocturnal sleep and memory encoding the following morning. We predicted that both exercise interventions would alter sleep compared to a control condition, resulting in improved memory encoding the next morning.

## Materials and methods

### Participants

Forty participants (19 female, 21 male, see Table 1 for demographics) completed the three sessions (control, high-intensity interval training [HIIT], and moderate-intensity continuous training [MICT]) of this within-subject design with counterbalanced order (i.e., a total of 120 experimental sessions were conducted). The inclusion criteria required participants to be healthy, physically active (engaging in at least 90 minutes of moderate-intensity exercise per week), non-smoking, native German speakers aged 18-35 years. Additionally, participants needed to have regular sleep-wake cycles (excluding shift workers), non-extreme chronotypes (bedtime between 22:00 and 01:00 and wake-up time between 06:00 and 09:00), and a Body Mass Index (BMI) below 30 kg/m². Participants with medical conditions or using medications or drugs that affect the nervous system or learning ability, as well as participants clinically diagnosed with a sleep, psychiatric/psychological and/or neurological disorder were excluded. The determination of our sample size was primarily influenced by the available resources, including time and budget constraints^45^. Additionally, we conducted a sensitivity analysis using G*Power version 3.1 to ascertain the smallest effect size that could be detected with 80% power (1 − β) in our preregistered analysis with a sample size of 40. The sensitivity analysis indicated that the minimum effect size we could detect was dz = 0.45, which we considered informative.

**Table 1:** Demographic variables.

Characteristics	All (39) Mean (SD)	All (39) Range	Female (18) Mean (SD)	Male (21) Mean (SD)
Age	25.18 (4.29)	[19–35]	25.17 (4.58)	25.19 (4.14)
BMI (kg/m^2^)	22.48 (2.67)	[17.4–29.4]	21.57 (2.68)	23.26 (2.46)
VO_2max_ (ml/min/kg)	49.44 (10.90)	[25–69]	43.33 (9.69)	54.67 (9.15)
IPAQ MET	4618 (2489)	[612–11118]	4845 (2846)	4424 (2190)
PSQI score	4.64 (1.94)	[0–8]	4.94 (1.92)	4.38 (1.96)

Before starting the study, participants were screened via an online questionnaire, which addressed demographic characteristics, chronotype (Morningness-Eveningness Questionnaire^46^, subjective sleep quality^47^, and general health condition^48^. This information was not subsequently used in any analysis.

One female participant was subsequently excluded because she did not adhere to our instructions in the control condition (exercised in the morning before the memory task). Demographics were calculated without this participant because she was excluded from all conditions. Data from two HIIT conditions were excluded (1 female, 1 male): one due to technical problems with the online task, and the other because the participant overslept. Data from four MICT sessions were also excluded (3 female, 1 male): two participants overslept, one exercised before the memory task, and one did not finish the memory task. Consequently, the final sample sizes for this within subject-design were 39 for the control condition, 37 for the HIIT condition, and 35 for the MICT condition (the statistical analysis allowed for missing data points, see section Data Analyses).

The study was approved by the local ethics committee of Heidelberg University and all participants gave written informed consent prior to participation in the study. All methods were carried out in accordance with relevant guidelines and regulations. This study was preregistered on the OSF (https://osf.io/ujs8h) and any deviations from the preregistered analyses are indicated below.

### Procedure

**Fig. 1:**
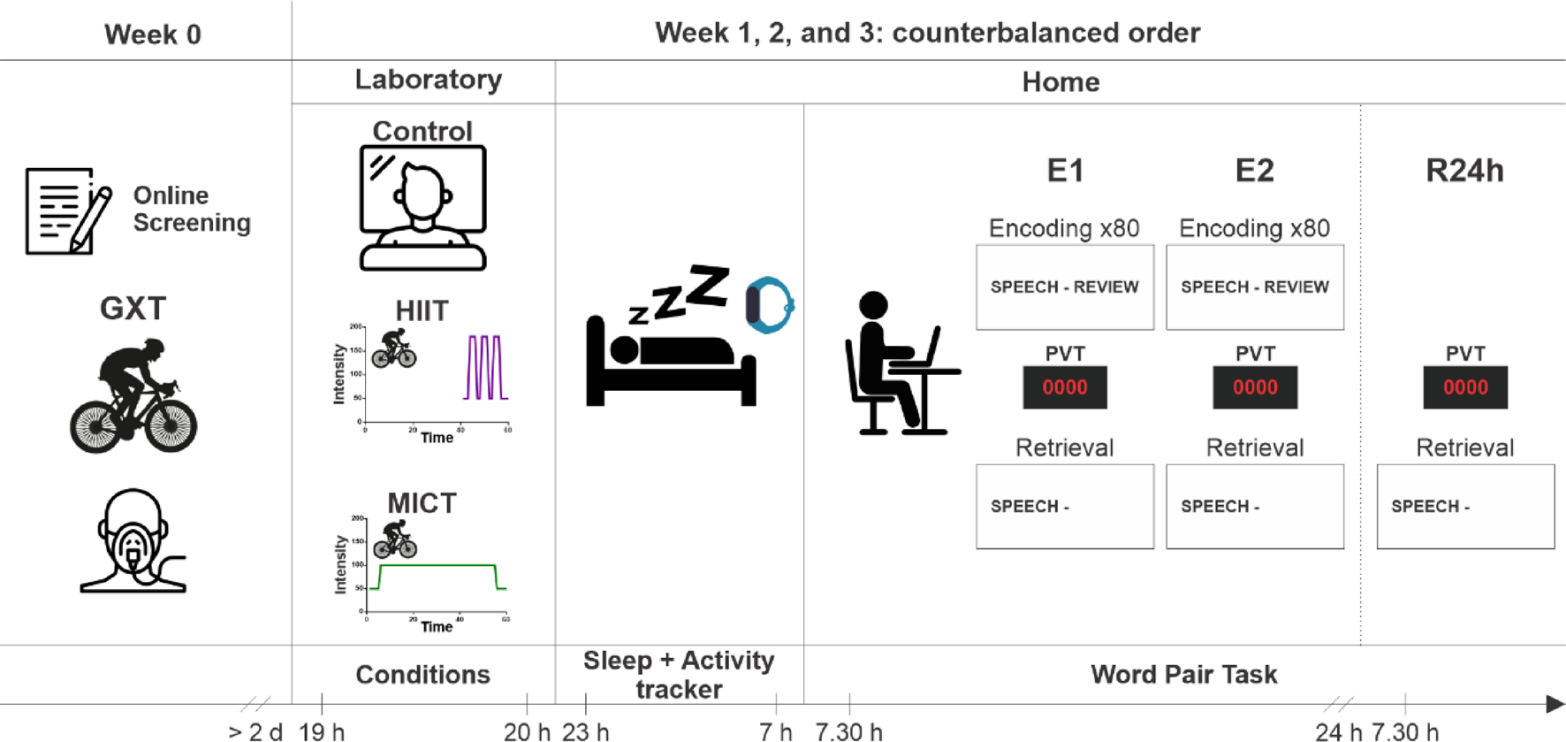
Experimental protocol. Participants first completed the graded exercise test (GXT). At least two days later, they began the first experimental condition (control, HIIT: high-intensity interval training or MICT: moderate-intensity continuous training). After completing the assigned condition, participants went home wearing the activity tracker and slept for eight hours. The following morning, 30 min after waking, they performed the memory and vigilance tasks (E1 and E2). Another twenty-four hours later, they completed the final retrieval session (R24h). The protocol was repeated for the two other conditions over the next consecutive weeks, with the order of conditions counterbalanced. The times shown are illustrative, as the protocol was adapted to the participants’ usual bedtime; however, the times between the different steps of the protocol remained the same. This cover has been designed using resources from Flaticon.com.

Participants visited the lab four times (Fig. 1). During the first visit, a graded exercise test (GXT) was performed to measure cardiorespiratory fitness and set exercise intensity levels for the experimental days. At least two days later, participants began the experimental conditions. We used a within-subject design, meaning participants took part in the control, HIIT, and MICT conditions in a counterbalanced order.

In each condition, participants arrived four hours before their usual bedtime. They performed the assigned condition for one hour, then took a 5-min shower, put on a wrist-worn actigraphy device, and went home. Participants were instructed to go to bed at their usual time, i.e., approximately three hours after finishing the condition in the laboratory, and to sleep for eight hours.

The next morning, they woke up, ate a provided granola bar, and approximately half an hour after waking, they completed the Stanford Sleepiness Scale^49^ and the St. Mary’s Sleep Questionnaire^50^. Immediately afterwards, they performed the memory and vigilance tasks online (see below). After finishing, they continued with their daily routines. The following morning, i.e., approximately 36 h after the exercise condition, they performed the vigilance task and the retrieval part of the memory task one more time. After completing the last test, actigraphy data collection was stopped.

Participants returned to the laboratory on the same day of the week for the two other conditions. During these weeks, they were instructed to keep their normal physical activity and sleep habits, but were not allowed to exercise or nap on the experimental days. In addition, participants were instructed not to consume caffeinated beverage after 2 p.m on these days. All this information was controlled by means of a questionnaire before starting the memory task.

### Interventions

*Control* Participants in the control condition watched a TV documentary for 60 min. We selected three episodes from the "Our Green Planet" series by Terra X (in German). The specific episode viewed during each session was also counterbalanced across conditions.

*High-intensity interval training (HIIT)* In this condition, participants watched a documentary for 40 min, and then performed the exercise intervention while continuing to watch the remaining 20 min. The exercise was performed on a cycle ergometer and began with a 3-min warm-up at 50 W. This was followed by three times 3-min intervals of cycling at 90% of their W_max_, interspersed with two 2-min intervals at 25% of W_max_. The session concluded with a 4-min cool-down phase at 50 W.

*Moderate-intensity continuous training (MICT)* In this condition, participants cycled continuously for 60 min at a moderate intensity. The session included a 5-min warm-up starting at 50 W, with the load progressively increasing to the target intensity. The main 50-min cycling workout maintained the participants’ heart rate between 60 and 70% of their HR_max_, determined during the GXT. Participants watched a TV documentary on a screen in front of them while exercising.

In both exercise conditions, participants were instructed to maintain a pedaling rate of at least 70 rpm throughout the protocol and were allowed to drink water ad libitum. They were also instructed to drink an isotonic gel. Heart rate was continuously recorded, and the rate of perceived exertion (RPE) was assessed with the Borg scale^51^.

### Memory and vigilance tasks

*Word Pair Task* This task was designed to test declarative learning performance using slightly associated word pairs. We created three different lists, each containing 80 word pairs, equivalent in difficulty and used in previous research^52^. The task was performed online in the participant’s home using JsPsych^53^. Each word pair from a list was displayed sequentially on the computer screen for 4 s, with a 1-s interval between pairs, followed by a 3-min version of the psychomotor vigilance task to buffer recency effects (PVT, explained below). After completing the task, the first retrieval session took place, where the cue word (first word in a pair) appeared and the participant had to type in the second target word (encoding performance, E1). This procedure was repeated a second time (E2), with the presentation time for each word pair reduced to 2 s. The next morning, participants performed the PVT again, followed by a recall session (24-h delayed retrieval, R24h). No feedback was provided in any of the retrieval phases, meaning that participants only had the opportunity to learn the word pairs twice.

*Psychomotor Vigilance Task (PVT)* This task measures participant’s average reaction speed as an indicator of vigilance^54^. The 3-min test required participants to press the space bar of their computer as soon as a bright red millisecond timer appeared on the computer screen, starting from 0000 ms. The subject’s reaction time was displayed immediately after pressing the space bar. The mean reaction speed (1/reaction time in ms) in E1, E2 and R24h was analyzed for each participant in each condition.

### Sleep data

Sleep data was recorded using activity monitors (GT9X Link, Actigraph Corp, Pensacola, Florida, USA) and analyzed with ActiLife 6 (Actigraph Corp, Pensacola, Florida, USA) software. From these monitors we obtained latency: time awake after bed onset; efficiency: number of sleep minutes divided by the total number of minutes the subject was in bed; total time in bed (TTB): the total number of minutes in bed; total sleep time (TST): the total number of minutes scored as “asleep”; wake after sleep onset (WASO): the total number of minutes the subject was awake after sleep onset; awakenings: the number of different awakening episodes; average awakening: the average length, in minutes, of all awakening episodes. Bedtimes were manually entered based on participants’ reports. To compare self-reported bedtimes across conditions, times were converted to numerical values relative to 00:00 h (e.g., 23:45 h = − 0.25). These transformed values were used in the analysis. However, for ease of interpretation, means and standard deviations are reported in hour format in the results table.

### Graded exercise test (GXT)

Participants underwent a GXT on a cycle ergometer (Ergoline GmbH, Ergoselect 5, Bitz Germany) with gas exchange measurement (CORTEX Biophysik GmbH, Metalyzer^®^ 3B, Leipzig, Germany) to assess baseline fitness level, including maximal oxygen uptake capacity (VO_2max_) and maximal power output (W_max_), as well as exercise response parameters (heart rate and blood pressure). The protocol consisted of a 3-min warm-up at 50 W, followed by stepwise increments of 15 W for women and 20 W for men every minute until subjective exhaustion, and concluded with a 5-min cool-down phase at 50 W afterwards^15,55^. Participants were instructed to keep a pedaling rate of at least 70 rpm throughout the protocol. In addition to the objective measurements, subjective RPE was assessed with the Borg scale.

### Data analyses

Data were analyzed using R version 4.4.0. Initially, we conducted the pre-registered analyses, i.e., paired t-tests to compare memory performance between the control condition and both the HIIT and MICT conditions at E1. Effect sizes are reported as Cohen’s *d*. We also performed a Pearson’s correlation to assess the relationship between sleep efficiency and memory performance.

To leverage all available data, including incomplete cases, we re-analyzed the data using (generalized) linear mixed models ((G)LMMs), which offer increased statistical power and provide a more robust analysis of repeated measures. These models are particularly well-suited for handling the dependencies inherent in such designs, as they can incorporate random intercepts to account for individual differences and, where appropriate, random slopes to model variability in participants’ responses across conditions^56^. These (G)LMMs were also used for the exploratory analyses. We implemented (G)LMMs using the lme4 package in R (version 1.1.35,^57^), and *p*-values were computed using the lmerTest package (version 3.1.3,^58^), applying Satterthwaite’s degrees of freedom method. For LMMs (only for the sleep and PVT data, where data was not binary), reported results include *t*-values, degrees of freedom, and *p*-values. GLMMs were used to evaluate the memory data at the trial level (i.e., unaggregated), since these were binary (correct vs. incorrect). Analyzing these data without previous aggregation allowed us to include random effects of the items and thus account for variance in the data stemming from idiosyncratic features of the individual word pairs. For GLMMs, reported results include *b*-coefficients on the log-odds scale, standard errors (SE), *z*-values, and *p*-values. All predictors were dummy coded: conditions (reference level: control), test sessions (reference level: E1), encoding time (reference level: early), and performance levels (reference level: low-performers). In our model equations, (…|subject) and (…|item) denote random effects by participants and individual word pairs, respectively. For each analysis, we first tried to fit a maximal model^56^. In cases where the model failed to converge, we addressed the issue by initially simplifying the random effects structure, specifically by removing interaction terms in the random slopes. For models resulting in singular fits, we further examined the correlation estimates among random effects. Random effects terms with correlations approaching ± 1 were identified and removed to achieve a well-specified and interpretable model. Once the best-fitting model was identified, we conducted model comparisons to determine whether a simpler model without the interaction term between the fixed effects provided a fit comparable to the more complex model that included the interaction. These comparisons were performed using the ANOVA function, relying on the likelihood ratio test (LRT) and following the recommendation of Matuschek et al.^59^, we used a significance threshold of α_LRT_ = 0.2.

To further explore the effects of experimental conditions and their interactions, as well as to obtain pairwise comparisons, we used estimated marginal means using the emmeans package in R (version 1.10.5,^60^). Because the main effects in models with interaction terms pertain only to the reference levels, we report the full model output (including these effects) in the Supplementary Material, while presenting the results from the pairwise comparisons in the main Results section. Pairwise comparisons are reported as estimated marginal means (EMMs) on the log-odds scale, along with their standard errors (SE), *z*-values, and *p*-values.

## Results

### Memory results

In our preregistered analysis approach, the control and HIIT conditions did not differ regarding the amount of word pairs recalled during the first morning learning session (t-test comparing encoding performance at E1, *t(36)* = 1.50, *p* = 0.143, Cohen’s *d* = 0.25, 95% CI [− 0.08, 0.57]). Similarly, the control and MICT conditions at E1 also did not differ regarding the amount of correct word pairs (*t(34)* = 0.23, *p* = 0.822, Cohen’s *d* = 0.04, 95% CI [− 0.29, 0.37]).

As indicated in the Data Analyses section we also used more powerful statistical methods and conducted a generalized linear mixed model that included binary data for each item during all measurement points (predictor *test*: E1, E2, and R24h) and for all participants (including those with missing data points as detailed in the methods section). The maximal-fitting GLMM for this analysis with a logit link function was: *memory performance (0 or 1)* ~ condition * test + *(1* + *condition | subject)* + *(1* + *condition | item)*. We compared this full model to a reduced model without the interaction term. The likelihood ratio test indicated no significant improvement in model fit (*χ*^2^(4) = 1.53, *p* = 0.821). Consequently, the final model used for inference was: *memory performance (0 or 1)* ~ *condition* + *test* + *(1* + *condition | subject)* + *(1* + *condition | item)*.

**Fig. 2:**
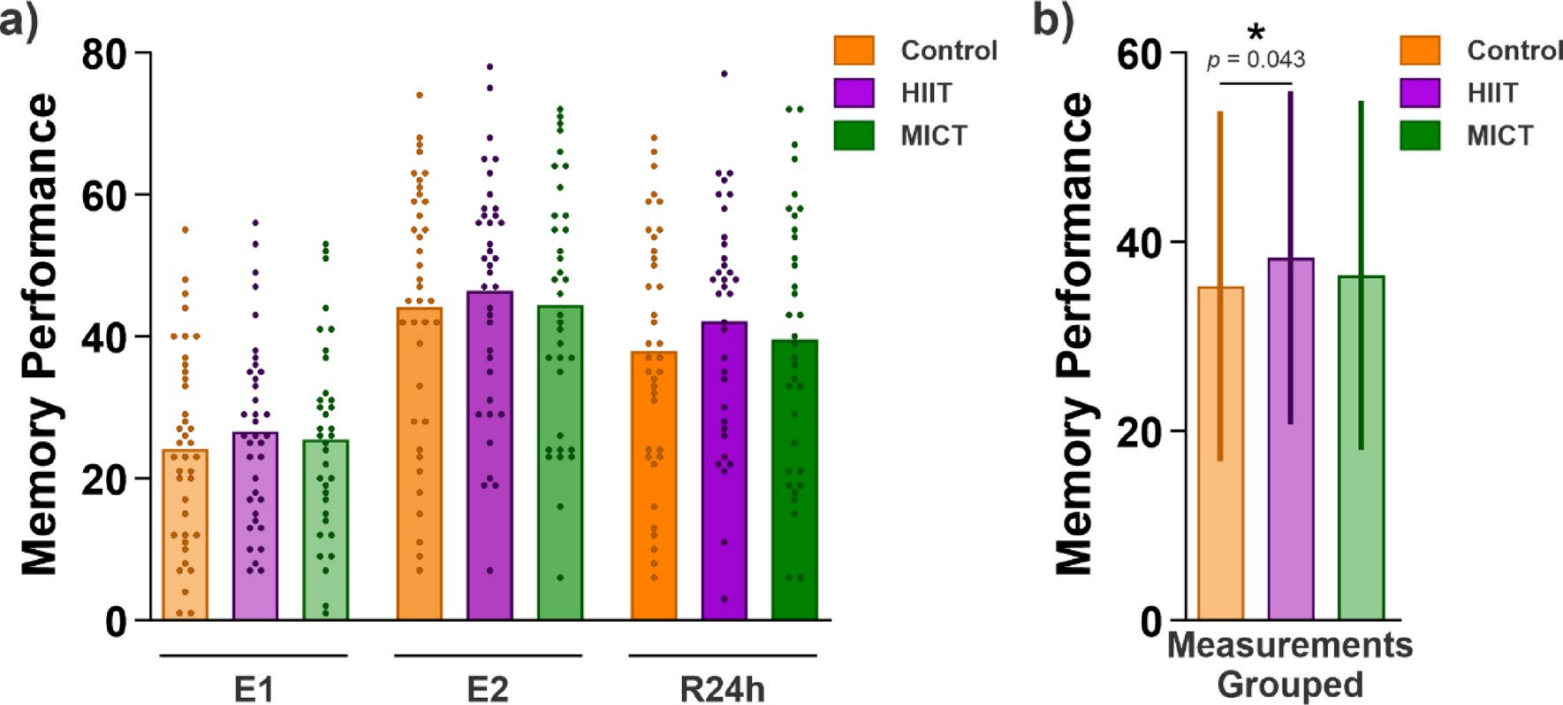
Positive effect of HIIT on memory performance. (**a**) Memory performance (i.e., the number of correctly recalled word pairs in each test at E1, E2 and R24h), is shown as the mean for each condition (Control, HIIT, and MICT) plus the performance of individual participants represented by dots. (**b**) The measurements across testing time points were averaged for each condition. The performance is shown as the mean ± SD, note that the effect of interest is within-subject. The generalized linear mixed model revealed that participants in the HIIT condition recalled more word pairs than in the control condition. Asterisks represent significance at α = 0.05.

The results indicated overall that participants in the HIIT condition recalled more word pairs than in the control condition (*b* = 0.27, *SE* = 0.13, *z* = 2.02, *p* = 0.043, Fig. 2). In contrast, participants in the MICT condition did not recall significantly more (or less) word pairs than in the control condition (*b* = 0.07, *SE* = 0.15, *z* = 0.46, *p* = 0.647). In test sessions E2 and R24h, participants recalled significantly more word pairs than in test session E1 (E2: *b* = 1.50, *SE* = 0.04, *z* = 38.11, *p* < 0.001; R24h: *b* = 1.10, *SE* = 0.04, *z* = 28.05, *p* < 0.001).

### Sleep results

We evaluated the effect of exercise on sleep parameters measured by actigraphy. The best-fitting LMM used for analyzing each sleep variable was specified as: *sleep variable* ~ *condition* + *(1 | subject)*. The results for the first night (the night after the intervention and before E1 and E2) revealed that participants spent approximately 8 h in bed as instructed, with no significant differences in time spent in bed between the control condition and both the HIIT and MICT conditions (Table 2, TTB). In addition, they spent approximately 6.5 h sleeping according to sleep latency and awakenings recorded by the actigraphy device, with no significant differences between conditions (Table 2, TST, WASO, # awakenings, and Avg. awakenings). No significant differences were found in any other sleep parameters between the control condition and the HIIT and MICT conditions (latency, efficiency and self-reported bedtime). Results for the second night’s sleep (before R24h) are provided in the supplementary materials (Supplementary Table S1).

**Table 2:** Sleep variables across experimental conditions.

	Control		HIIT			MICT		
Variable	Mean	SD	Mean	SD	*p*-value	Mean	SD	*p*-value
Bedtime (hh:mm)	23:17	00:50	23:19	00:43	0.871	23:25	00:55	0.116
Latency (min)	12.2	12.7	9.5	8.3	0.263	9.9	11.3	0.308
Efficiency (%)	79.9	7.5	80.0	7.9	0.951	79.8	7.0	0.888
TTB (min)	486	33	492	38	0.357	489	34	0.281
TST (min)	388	48	394	56	0.546	391	51	0.447
WASO (min)	85.1	38.3	88.3	34.9	0.657	88.3	35.0	0.736
# awakenings	30.4	11.0	30.0	8.2	0.555	29.9	8.0	0.599
Avg awakenings (min)	2.9	1.0	3.1	1.4	0.331	3.0	1.3	0.472

Means and standard deviations (SD) for sleep variables (Bedtime, Latency, Efficiency, Total Time in Bed [TTB], Total Sleep Time [TST], Wake After Sleep Onset [WASO], number [#] of awakenings, and average awakenings) are shown for each condition. In addition, the *p*-values from the comparisons between control and both HIIT and MICT conditions are displayed.

We also conducted the pre-registered correlation analysis between sleep efficiency and the amount of word pairs recalled at time point E1. There was no significant correlation between these two variables (r = − 0.002, *p* = 0.981; see Supplementary Fig. S1).

### Exploratory results

**Fig. 3:**
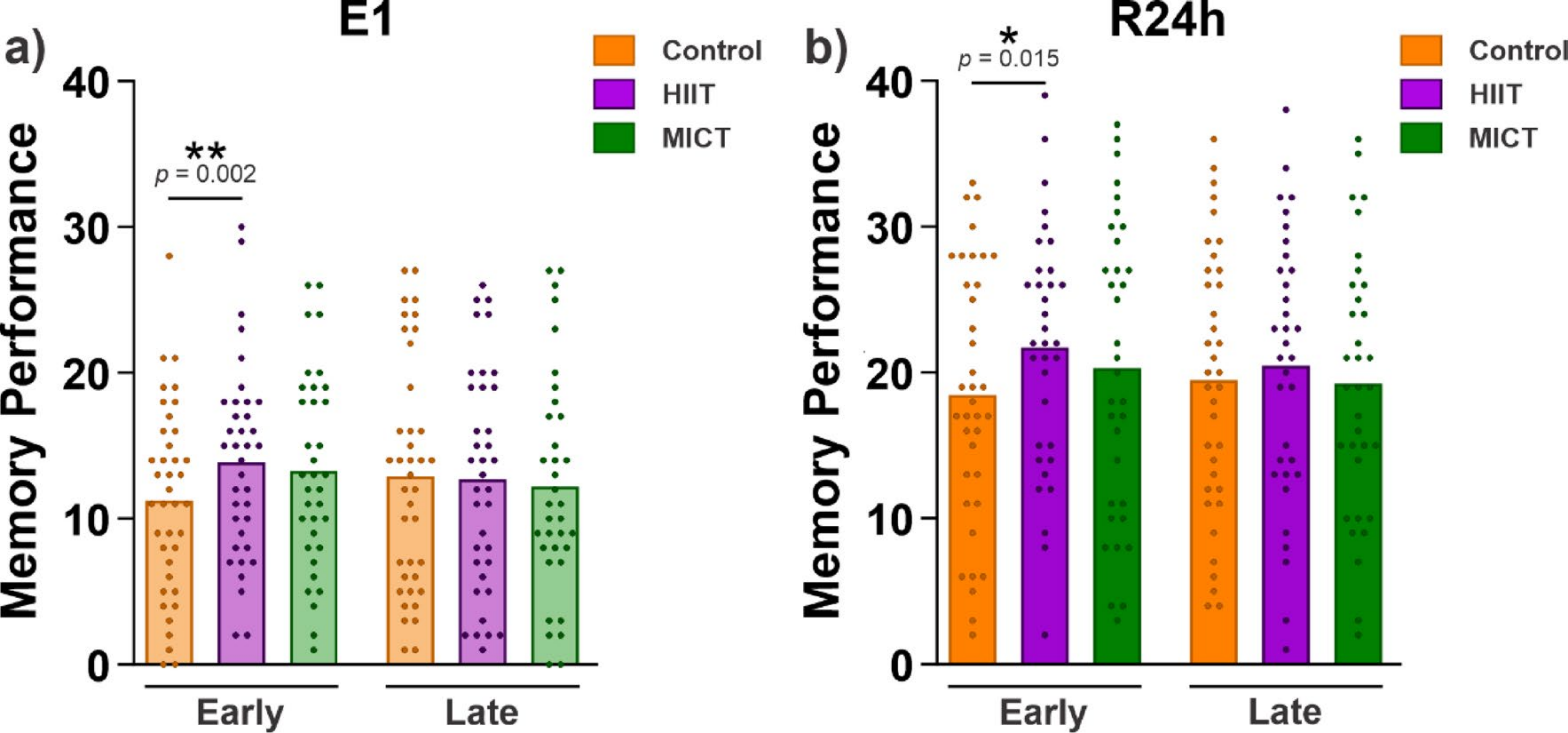
Participants’ memory performance across conditions, separated into first and second halves of encoding. Memory performance for the first (early) and second (late) halves of words encoded in E1 during the (**a**) E1 and (**b**) R24h tests. Data are shown as the mean for each condition and each dot represents the performance of individual participants. The generalized linear mixed models and the pairwise comparisons results from the estimated marginal means revealed that participants recalled more word pairs only from the first half in the HIIT condition than in the control condition in both tests: E1 and R24h. The results for E2 are provided in Supplementary Table S4 and S5. Asterisks represent significance at α = 0.05.

We conducted two exploratory analyses. First, we investigated whether exercise specifically influenced the initial phase of memory encoding, as would be expected according to the synaptic homeostasis hypothesis. To assess this, we divided the encoding phase into two halves: the first 40 word pairs (early encoding) and the second 40 word pairs (late encoding), analyzing memory performance for each encoding time separately (predictor *encoding time*: early and late). The maximal-fitting GLMM for this analysis with a logit link function was: *memory performance (0 or 1)* ~ condition * encoding time + *(1* + *condition* + *encoding time | subject)* + *(1 | item)*. We compared this full model to a reduced model without the interaction term. The likelihood ratio test indicated significant improvements in model fit (*χ*^2^(2) = 13.01, *p* = 0.001), and therefore, the interaction term was included. We first analyzed E1, as adding E2 and R24h to the analysis would make it hard to interpret due to the added interaction. The full table with model output for E1 is included in the supplementary material (Supplementary Table S2). The pairwise comparisons revealed that participants recalled significantly more word pairs from the first half (early) of word pairs encoded during the E1 test in the HIIT condition than in the control condition (*EMM* = *0.46, SE* = *0.14, z* = *3.35, p* = *0.002*, Fig. 3a), which was not the case for the second half (late: *EMM* = *0.07, SE* = *0.14, z* = *0.50, p* = *0.870*). No significant difference in the amount of word pairs correctly recalled was found for the early and late encoding between the control and MICT conditions (*early: EMM* = *0.26, SE* = *0.17, z* = *1.57, p* = *0.261; late: EMM* = − *0.16, SE* = *0.17, z* = *-0.95, p* = *0.609*). In addition, no significant difference in the amount of word pairs correctly recalled was found for the early and late encoding between the HIIT and MICT conditions (*early: EMM* = − *0.20, SE* = *0.14, z* = − *1.50, p* = *0.292; late: EMM* = *-0.23, SE* = *0.14, z* = − *1.64, p* = *0.227*).

Next, we analyzed memory performance in the R24h test for the same words encoded in the first and second halves of the initial encoding session (Fig. 3b). The pattern of results of this analysis was similar to that of the analysis in E1, and the full output table is reported in the supplementary material (Supplementary Table S3). The pairwise comparisons revealed that participants in the R24h test recalled significantly more word pairs only from the first half encoded (early) in the HIIT condition compared to the control condition (early: *EMM* = 0.43, *SE* = 0.16, *z* = 2.77, *p* = 0.015; late: *EMM* = 0.14, *SE* = 0.16, *z* = 0.87, *p* = 0.662). There was also no significant difference in the number of word pairs correctly recalled from the early and late encoding between the control and MICT conditions (early: *EMM* = *0.22, SE* = *0.18, z* = *1.19, p* = *0.459;* late: *EMM* = *-0.06, SE* = *0.18, z* = − *0.35, p* = *0.935*). In addition, no significant difference in the amount of word pairs correctly recalled was found for the early and late encoding between the HIIT and MICT conditions (*early: EMM* = − *0.22, SE* = *0.16, z* = − *1.40, p* = *0.342; late: EMM* = − *0.20, SE* = *0.16, z* = − *1.28, p* = *0.410*). A similar pattern of results was found for E2 (see Supplementary Table S4 and Supplementary Table S5).

**Fig. 4:**
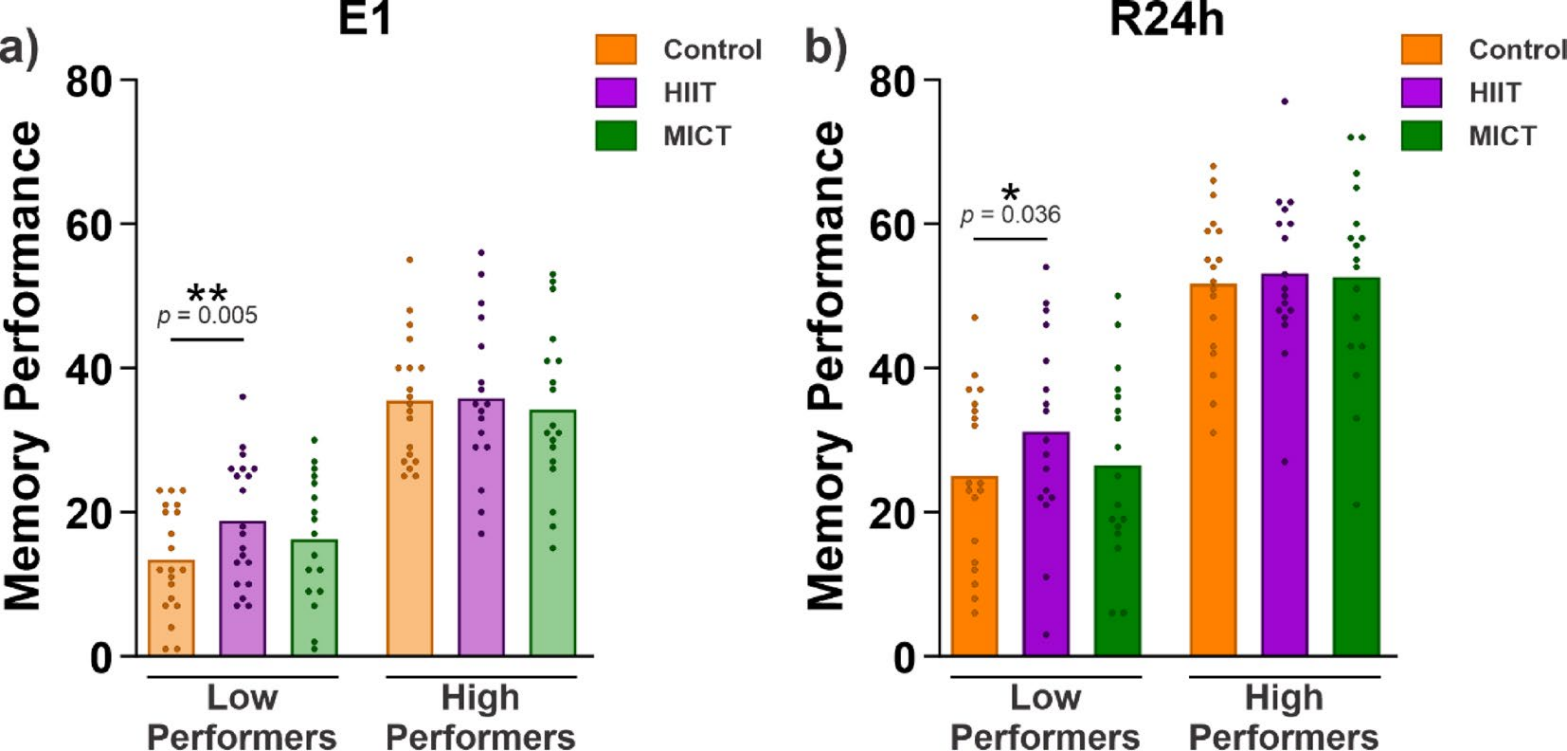
Participants’ memory performance across conditions, separated into low- and high- performers. Memory performance for low- and high-performing participants in the (**a**) E1 and (**b**) R24h tests. Data are shown as the mean for each condition and each dot represents the performance of individual participants. The generalized linear mixed models and the pairwise comparisons results from the estimated marginal means revealed that only low-performing participants recalled more word pairs in the HIIT condition than in the control condition in both E1 and R24h. Asterisks represent significance at α = 0.05. The statistical results for E2 are shown in the Supplementary Table S8 and S9.

In the second exploratory analysis, we tested whether exercise had different effects in participants with low and high memory performance. To examine this, we divided participants into low- and high-performing groups based on a post hoc median split of their memory performance in the control condition during E1 (predictor *performer*: low and high). The maximal-fitting GLMM for this analysis with a logit link function was: *memory performance (0 or 1)* ~ condition * performer + *(1* + *condition | subject)* + *(1 + performer | item)*. We compared this full model to a reduced model without the interaction term. The likelihood ratio test indicated significant improvements in model fit (*χ*^2^(2) = 4.50, *p* = 0.105), and therefore, the interaction term was included. The full table with model output for E1 is included in the supplementary material (Supplementary Table S6). The pairwise comparisons revealed that low-performing participants recalled significantly more word pairs in the HIIT condition than in the control condition (*EMM* = 0.51, *SE* = 0.16, *z* = 3.10, *p* = 0.005, Fig. 4a), which was not the case for high-performing participants (*EMM* = 0.01, *SE* = 0.16, *z* = 0.06, *p* = 0.998). Low-performing participants did not differ in the number of word pairs recalled in the control and MICT conditions (*EMM* = 0.24, *SE* = 0.22, *z* = 1.12, *p* = 0.502). The same pattern was observed for high-performing participants (*EMM* = − 0.12, *SE* = 0.20, *z* = − 0.60, *p* = 0.822). Additionally, low- and high-performing participants did not differ in the amount of words recalled in the HIIT and MICT conditions (low: *EMM* = − 0.27, *SE* = 0.17, *z* = − 1.55, *p* = 0.268; high: *EMM* = − 0.13, *SE* = 0.16, *z* = − 0.79, *p* = 0.709).

The pattern of results in the R24h test was similar to that of the analysis in E1, and the full output table is reported in the supplementary material (Supplementary Table S7). The pairwise comparisons revealed that only low-performing participants recalled significantly more word pairs in the HIIT than in the control condition in the R24h test (low: *EMM* = 0.46, *SE* = 0.19, *z* = 2.38, *p* = 0.046; high: *EMM* = 0.14, *SE* = 0.20, *z* = 0.71, *p* = 0.757, Fig. 4b). Neither low- nor high-performers differed in the number of words recalled between the control and MICT conditions (low: *EMM* = 0.13, *SE* = 0.24, *z* = 0.56, *p* = 0.841; high: *EMM* = 0.06, *SE* = 0.24, *z* = 0.26, *p* = 0.965). Additionally, low- and high-performing participants did not differ in the amount of words recalled in the HIIT and MICT conditions (low: *EMM* = − 0.32, *SE* = 0.20, *z* = − 1.62, *p* = 0.236; high: *EMM* = − 0.08, *SE* = 0.20, *z* = − 0.40, *p* = 0.917). A similar pattern of results was found in E2 (see Supplementary Table S8 and Supplementary Table S9).

### Control measures

#### Objective vigilance

Regarding our test of objective vigilance, we found no significant differences in reaction speed between the conditions in the PVT; however, participants were significantly slower in E2 compared to E1 across all conditions (*t*(73.48) = 3.94, *p* < 0.001). Detailed statistics are provided in the supplementary information (Supplementary Table S10).

In addition, we compared the reaction speed in E1, E2 and R24h for low and high-performers and found no significant differences between them (E1: *t*(39.64) = − 1.66, *p* = 0.104; E2: *t*(39.54) = − 1.55, *p* = 0.129; R24h: *t*(38.66) = − 1.23, *p* = 0.225).

#### Physical load

To quantify the internal training load experienced during each intervention, we calculated the training impulse (TRIMP) for each participant using Banister’s method^61^. Results indicated that the MICT condition elicited significantly higher TRIMP values compared to the HIIT condition (*t*(34.89) = 4.05, *p* < 0.001). The TRIMP calculation (Supplementary material, Eq. 1) and the mean values for each condition are provided in the Supplementary Information (Supplementary Table S11).

## Discussion

In this study, we investigated the effects of two evening exercise interventions on post-sleep memory encoding of word pairs and demonstrated the utility of high-intensity exercise for basic systems level research and for potential future applications to improve memory performance. To our knowledge, this is the first study of this kind. To achieve a larger sample size than has usually been reported in the fields of exercise and sleep^26,43,44,62^, we used a novel approach that combines online^53^ and in-lab experimental methodologies. Consistent with our preregistered hypothesis, our findings indicate that an acute bout of intense exercise (HIIT) three hours before bedtime significantly enhances memory performance the following morning. However, this effect only emerged when we employed more robust statistical methods (not preregistered). (G)LMMs are well-suited for data with hierarchical or crossed structures, as they allow for the inclusion of random intercepts and slopes to model variability across participants and items. By accounting for individual differences in both baseline performance and responses to experimental conditions, these models provide more accurate estimates of effects and variance components. This improves sensitivity to detect patterns in the data that might otherwise be obscured^56,63^. Contrary to our predictions, this improvement was not observed following an acute bout of moderate exercise (MICT). Actigraphy-derived sleep data did not show any significant changes to sleep due to the interventions. Exploratory analyses revealed that participants in the HIIT condition encoded the early items of the word pair task significantly better than in the control condition. The early items were also better recalled after 24 h. Furthermore, participants with lower encoding abilities exhibited improved memory encoding in the HIIT condition compared to the control condition, which extended to enhanced memory retention after 24 h. This effect was not present in high-performing participants, nor did we find evidence for such an effect in the MICT condition.

Previous studies have investigated how exercise administered immediately before memory encoding can impact memory performance^64,65^. However, these studies did not schedule sleep in-between to observe its impact on post-sleep memory encoding. Although we did not observe clear changes in sleep parameters using actigraphy it is tempting to speculate that the improvement in memory encoding can be explained by HIIT influencing sleep architecture in ways not detectable with this method. Unfortunately, we were unable to measure sleep via polysomnography to make an explicit link, however, according to the sleep and synaptic homeostasis hypothesis^5,66^, specific features of NREM sleep, such as SWA and spindles, play a crucial role in restoring encoding capacity^41–44^. Slow waves (activity in the 1–4 Hz range) facilitate synaptic downscaling, while spindles support hippocampal-to-neocortical transfer of reactivated information, both of which are linked to improved learning^2,5,7,66^. It is thus a plausible hypothesis that HIIT exerts its cognitive benefits via modulation of these sleep features, potentially through mechanisms such as exercise-induced thermoregulation changes that have been associated with increased SWA and spindle activity^26^. While previous studies have reported increases in N2 and SWS following high-intensity exercise^26–28,30^, further research using polysomnography would be needed to support this speculation.

Although we expected both exercise interventions to improve memory performance, only the high-intensity exercise led to a significant enhancement, whereas the moderate-intensity condition did not produce a comparable effect. Interestingly, the higher TRIMP values in the moderate-intensity condition did not appear to translate into greater effects on sleep. This is likely because besides physical load, acute metabolic and neurochemical responses, such as increased lactate production, elevated catecholamine levels, and enhanced brain-derived neurotrophic factor release, may play an important role in sleep modifications and are typically more pronounced following high-intensity exercise^10,67^. While some studies have reported increased SWA and SWS following moderate exercise^27,68,69^, these findings were based on small sample sizes (n < 10), and in some cases, the increases were not statistically significant. A potential explanation for our findings is that MICT may not have induced meaningful changes in sleep architecture sufficient to enhance memory encoding the following morning, although polysomnography data would be needed to demonstrate this. Interestingly, Aritake-Okada and colleagues’ study^26^ found significant increases in SWS following four sessions of moderate-intensity exercise in a sedentary population, highlighting the role of exercise volume and participant fitness. In addition, some studies have found that SWS elevation occurs only after high-intensity, but not moderate-intensity, exercise^62^. Further well-powered studies with polysomnography are needed to better understand how different exercise intensities affect sleep architecture and their subsequent impact on memory.

In studies measuring sleep with both actigraphy and polysomnography, exercise-induced changes, such as reduced REM sleep and increased non-REM sleep, were found only with polysomnography^70–72^. Although our study was run using a much larger sample (at least 35 subjects per condition, within-subjects, compared to fewer than 13 in previous studies) allowing to detect behavioral results with more precision this led to resource constraints and only allowed us to collect actigraphy and not polysomnographic data. Hence, it may be unsurprising that we did not find significant differences in any of the sleep variables measured with actigraphy. While this provides robust evidence that actigraphy lacks sensitivity to detect exercise-driven changes in sleep it does not necessarily indicate that HIIT has no measurable effect on sleep architecture, since REM and NREM sleep can only be reliably differentiated with polysomnography and this more involved technique allows identifying slow waves and sleep spindles. Notably, we did not observe any sleep disturbances resulting from the evening exercise interventions since sleep quality remained high, which is important to consider, when considering such interventions for broader use. In sum, polysomnography is needed to determine effects of exercise on sleep.

To gain further insight into the influence of exercise on morning encoding, we conducted two exploratory analyses. We found better early encoding in the HIIT group than in the control group. Notably, the enhanced early encoding of the HIIT group led to better memory performance of those early-encoded pairs also after 24 h. A possible explanation is that the second half of the word pairs retroactively interfered with the memory of the first half, a phenomenon observed in previous studies^73,74^. The HIIT intervention may counteract this interference by enhancing synaptic downscaling leading to more robust encoding of the early items and stronger traces as indicated by better memory performance. Second, we found that participants with lower encoding abilities showed improved memory encoding in the HIIT condition compared to the control condition, which also led to better memory performance in the 24-h delay test. Previous studies have examined the effects of sleep on memory consolidation in low- and high-performers, yielding mixed results: Diekelmann and colleagues^75^ found a positive effect of sleep only for low memory performers, while Tucker and Fishbein^76^ observed a positive effect only for high-performers. One study has shown that a closed-loop tACS intervention, designed to enhance slow-wave oscillations, can prevent retroactive interference compared to sham stimulation, particularly in poor encoders^77^. Although the protocol differs from ours, the HIIT intervention may similarly help low-encoders compensate poor encoding abilities. While these findings should be interpreted with caution, they are promising, as this simple intervention, potentially feasible for home use, could offer a practical way to help individuals with memory impairments improve their cognitive function.

Although our pre-registered analyses were not significant, the more powerful GLMMs, which utilized the full variance of the repeated measurements, proved to be sensitive in detecting differences. Improving the statistical analysis can be a reason to deviate from a preregistration^78^. Importantly, we preregistered our study to promote transparency and replicability, even though our investigation explored a novel hypothesis; we therefore place a decent amount of trust in our results that nonetheless need to be replicated independently in the future. We did not observe any effects on sleep parameters; therefore, our attribution of the behavioral effects on memory to the interaction between exercise and sleep remains a speculation until supported by polysomnography. Nonetheless, our innovative approach of combining in-lab and online experiments allowed us to include a larger sample size than usual, and compared two types of exercise interventions, optimizing the informative value of our data within the constraints of available resources^45^.

In conclusion, we demonstrate a positive effect of an acute evening bout of HIIT on post-sleep memory performance, especially for individuals with lower encoding abilities. This is the first study to explore this specific interaction, laying important groundwork for future research to further investigate the mechanisms underlying these findings. Notably, this approach offers a simple way that might be valid to modify sleep and assess its impact on brain plasticity and memory encoding, which may offer new insights into the mechanisms of the synaptic renormalization. Moreover, these findings may establish the basis for practical interventions readily applicable in populations suffering from both sleep and memory disorders.

## Electronic supplementary material

Below is the link to the electronic supplementary material.

Supplementary Material 1

## Data Availability

All data (10.23668/psycharchives.16463) and code (10.23668/psycharchives.16464) are publicly available at the PsychArchives of the Leibniz Institute of Psychology (ZPID).
