# Supplementary material for: High intensity exercise before sleep boosts memory encoding the next morning
Source: Sci Rep. 2025 Jul 1;15:20388. doi: 10.1038/s41598-025-07880-z (PMC12217599; doi:10.1038/s41598-025-07880-z)
Supplement: Supplementary file 1 — Supplementary Material 1 [file 41598_2025_7880_MOESM1_ESM.pdf]

Supplementary Material for "**High intensity exercise before sleep boosts memory encoding the next morning**"

Daniela Ramirez Butavand[a,b,f*], Juliane Nagel[b,c,d,e,f], Gordon B. Feld[b,c,d,e,f,1] & Simon Steib[a,g,1]

[a]Human Movement, Training and Active Aging Department, Institute of Sports and Sports Science, Heidelberg University, Heidelberg, Germany.

[b]Clinical Psychology, Central Institute of Mental Health, Medical Faculty Mannheim, Heidelberg University, Mannheim, Germany.

[c]Addiction Behavior and Addiction Medicine, Central Institute of Mental Health, Medical Faculty Mannheim, Heidelberg University, Mannheim, Germany.

[d]Psychiatry and Psychotherapy, Central Institute of Mental Health, Medical Faculty Mannheim, Heidelberg University, Mannheim, Germany.

[e]Psychological Institute, Heidelberg University, Heidelberg, Germany.

[f]German Center for Mental Health (DZPG).

[g]Network Aging Research, Heidelberg University

[1]GF and SS contributed equally to this work

*daniela.ramirez@issw.uni-heidelberg.de

Supplementary analysis 1

The sleep results for the second night (before R24h) can be found in the Supplementary Table S1. The best-fitting model for each sleep variable was: *sleep variable ~ condition + (1* **|** *subject)*.

Supplementary Table S1: **Sleep Variables for the second night (before R24h).** The mean and standard deviation (SD) values for each sleep variable (Bedtime, Latency, Efficiency, Total Time in Bed [TTB], and Total Sleep Time [TST], Wake After Sleep Onset [WASO], number [#] of awakenings, and average awakenings) are shown for each condition. In addition, the *p*-values from the comparisons between Control and both HIIT and MICT conditions are displayed.

	**Control**		**HIIT**			**MICT**		
**Variable**	**Mean**	**SD**	**Mean**	**SD**	***p*-value**	**Mean**	**SD**	***p*-value**
**Bedtime** (hh:mm)	23:45	01:21	23:48	01:06	0.778	23:43	01:11	0.925
**Latency** (min)	12.4	13.9	10.5	15.7	0.581	15.7	16.2	0.364
**Efficiency** (%)	79.4	8.5	78.5	9.7	0.680	77.8	7.9	0.532
**TTB** (min)	485	66	484	63	0.816	487	66	0.878
**TST** (min)	385	62	379	64	0.570	378	63	0.821
**WASO** (min)	88.2	43.4	92.6	42.5	0.493	94.6	48.9	0.805
**#awakenings**	28.5	12.3	31.9	9.8	0.114	31.4	10.6	0.061
**Avg. Awakenings** (min)	3.2	1.3	2.9	1.0	0.296	3.0	1.2	0.130

Supplementary analysis 2

We performed the pre-registered correlation between sleep efficiency and memory performance. There was no significant correlation between these two variables (*r* = -0.002, *p* = 0.981, Supplementary Fig. S1).

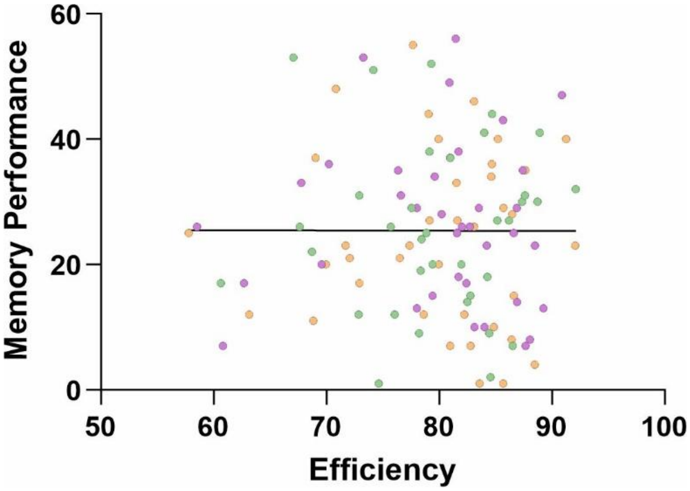

Supplementary Figure S1: **Correlation between memory performance and sleep efficiency.** Orange dots belong to the control condition, purple dots belong to the HIIT condition and green dots to the MICT condition.

Supplementary analysis 3

For analyzing the effect of exercise on the different phases of encoding (early and late) we ran the following best-fitting model: *memory performance (0 or 1) ~ condition * encoding time + (1 + condition + encoding time* | *subject) + (1* | *item).* The full model outputs are reported in Supplementary Table S2 (E1) and Supplementary Table S3 (R24h). The results of additional pairwise comparisons of the E1 and R24h analyses can be found in the main manuscript.

The analysis for E2 was run with the following model: *memory performance (0 or 1) ~ condition * encoding time + (1 + condition* | *subject) + (1* | *item)* because the model used for the other two analyses presented boundary singular fit in the E2 data. The full model output for E2 are reported in Supplementary Table S4 and the results of additional pairwise comparisons of E2 in Supplementary Table S5.

Supplementary Table S2: **Full model output of the analysis including early and late encoding in E1 test.** The reference level for the predictor "condition" was control and for the predictor "encoding" (referred to as "encoding time" in the model) was early.

Effect	Group	Term	b	SE	z	*p*-value
fixed	–	(Intercept)	-1.26	0.18	-7.02	< .001
fixed	–	conditionHIIT	0.46	0.14	3.35	< .001
fixed	–	conditionMICT	0.26	0.17	1.57	.117
fixed	–	encodinglate	0.15	0.11	1.38	.167
fixed	–	conditionHIIT:encodinglate	-0.39	0.13	-3.07	.002
fixed	–	conditionMICT:encodinglate	-0.42	0.13	-3.20	.001
random	item	sd (Intercept)	0.93	–	–	–
random	subject	sd (Intercept)	0.96	–	–	–
random	subject	cor (Intercept).conditionHIIT	-0.57	–	–	–
random	subject	cor (Intercept).conditionMICT	-0.48	–	–	–
random	subject	cor (Intercept).encodinglate	0.71	–	–	–
random	subject	sd conditionHIIT	0.64	–	–	–
random	subject	cor conditionHIIT.conditionMICT	0.72	–	–	–
random	subject	cor conditionHIIT.encodinglate	-0.53	–	–	–
random	subject	sd conditionMICT	0.83	–	–	–
random	subject	cor conditionMICT.encodinglate	0.00	–	–	–
random	subject	sd encodinglate	0.35	–	–	–

Supplementary Table S3: **Full model output of the analysis including early and late encoding in R24h test.** The reference level for the predictor "condition" was control and for the predictor "encoding" (referred to as "encoding time" in the model) was early.

Effect	Group	Term	b	SE	z	*p*-value
fixed	–	(Intercept)	-0.23	0.22	-1.04	.297
fixed	–	conditionHIIT	0.43	0.16	2.77	.006
fixed	–	conditionMICT	0.22	0.18	1.19	.234
fixed	–	encodinglate	0.09	0.09	0.92	.358
fixed	–	conditionHIIT:encodinglate	-0.30	0.13	-2.26	.024
fixed	–	conditionMICT:encodinglate	-0.28	0.13	-2.09	.037
random	item	sd (Intercept)	1.17	–	–	–
random	subject	sd (Intercept)	1.19	–	–	–
random	subject	cor (Intercept).conditionHIIT	-0.31	–	–	–
random	subject	cor (Intercept).conditionMICT	-0.22	–	–	–
random	subject	cor (Intercept).encodinglate	0.57	–	–	–
random	subject	sd conditionHIIT	0.71	–	–	–
random	subject	cor conditionHIIT.conditionMICT	0.64	–	–	–
random	subject	cor conditionHIIT.encodinglate	-0.41	–	–	–
random	subject	sd conditionMICT	0.88	–	–	–
random	subject	cor conditionMICT.encodinglate	0.16	–	–	–
random	subject	sd encodinglate	0.13	–	–	–

Supplementary Table S4: **Full model output of the analysis including early and late encoding in E2 test.** The reference level for the predictor "condition" was control and for the predictor "encoding" (referred to as "encoding time" in the model) was early.

**Effect**	**Group**	**Term**	**b**	**SE**	**z**	***p*-value**
fixed	–	(Intercept)	0.23	0.23	0.99	.321
fixed	–	conditionHIIT	0.35	0.15	2.34	.019
fixed	–	conditionMICT	0.11	0.16	0.69	.489
fixed	–	encodinglate	0.05	0.09	0.61	.545
fixed	–	conditionHIIT:encodinglate	-0.25	0.13	-1.91	.057
fixed	–	conditionMICT:encodinglate	-0.20	0.13	-1.50	.133
random	item	sd (Intercept)	1.11	–	–	–
random	subject	sd (Intercept)	1.29	–	–	–
random	subject	cor (Intercept).conditionHIIT	-0.39	–	–	–
random	subject	cor (Intercept).conditionMICT	-0.35	–	–	–
random	subject	sd conditionHIIT	0.72	–	–	–
random	subject	cor conditionHIIT.conditionMICT	0.60	–	–	–
random	subject	sd conditionMICT	0.79	–	–	–

Supplementary Table S5: **Pairwise comparisons results from the estimated marginal means.** Pairwise comparisons for the analysis of the effect of exercise on the different phases of encoding (early and late) on E2 data.

**Contrast**	**EMM**	**SE**	***z***	***p*-value**
**HIIT – Control** (early)	0.35	0.15	2.34	0.051
**MICT – Control** (early)	0.11	0.16	0.69	0.769
**MICT – HIIT** (early)	-0.24	0.15	-1.60	0.246
**HIIT – Control** (late)	0.11	0.15	0.71	0.756
**MICT – Control** (late)	-0.08	0.16	-0.51	0.865
**MICT – HIIT** (late)	-0.19	0.15	-1.28	0.406

Supplementary analysis 4

For analyzing the effect of exercise on low- and high-performing participants we ran the following best-fitting model: *memory performance (0 or 1) ~ condition * performer + (1 + condition* | *subject) + (1 + performer* | *item).* The full model output for E1 test is reported in the Supplementary Table S6 and for R24h test in the Supplementary Table S7. The results of additional pairwise comparisons of the E1 and R24h analyses can be found in the main manuscript.

The full model output for E2 is reported in Supplementary Table S8 and additional pairwise comparisons of the E2 analysis can be found in Supplementary Table S9.

Supplementary Table S6: **Full model output of the analysis including low- and high-performing participants in the E1 test**. The reference level for the predictor "condition" was control and for the predictor "performer" was low.

Effect	Group	Term	b	SE	z	***p*-value**
fixed	–	(Intercept)	-1.96	0.17	-11.62	< .001
fixed	–	conditionHIIT	0.51	0.16	3.10	.002
fixed	–	conditionMICT	0.24	0.22	1.12	.263
fixed	–	performerhigh	1.67	0.22	7.55	< .001
fixed	–	conditionHIIT:performerhigh	-0.50	0.23	-2.17	.030
fixed	–	conditionMICT:performerhigh	-0.36	0.29	-1.23	.219
random	item	sd (Intercept)	0.86	–	–	–
random	item	cor (Intercept).performerhigh	0.55	–	–	–
random	item	sd performerhigh	0.22	–	–	–
random	subject	sd (Intercept)	0.61	–	–	–
random	subject	cor (Intercept).conditionHIIT	-0.46	–	–	–
random	subject	cor (Intercept).conditionMICT	-0.39	–	–	–
random	subject	sd conditionHIIT	0.57	–	–	–
random	subject	cor conditionHIIT.conditionMICT	0.69	–	–	–
random	subject	sd conditionMICT	0.78	–	–	–

Supplementary Table S7: Full model output of the analysis including low- and high-performing participants in the R24h test. The reference level for the predictor "condition" was control and for the predictor "performer" was low.

**Effect**	**Group**	**Term**	**b**	**SE**	**z**	***p*-value**
fixed	–	(Intercept)	-1.06	0.20	-5.33	< .001
fixed	–	conditionHIIT	0.46	0.19	2.38	.017
fixed	–	conditionMICT	0.13	0.24	0.56	.575
fixed	–	performerhigh	1.86	0.27	6.90	< .001
fixed	–	conditionHIIT:performerhigh	-0.32	0.28	-1.15	.251
fixed	–	conditionMICT:performerhigh	-0.07	0.34	-0.21	.832
random	item	sd (Intercept)	1.04	–	–	–
random	item	cor (Intercept).performerhigh	0.73	–	–	–
random	item	sd performerhigh	0.32	–	–	–
random	subject	sd (Intercept)	0.77	–	–	–
random	subject	cor (Intercept).conditionHIIT	-0.23	–	–	–
random	subject	cor (Intercept).conditionMICT	-0.27	–	–	–
random	subject	sd conditionHIIT	0.69	–	–	–
random	subject	cor conditionHIIT.conditionMICT	0.65	–	–	–
random	subject	sd conditionMICT	0.89	–	–	–

Supplementary Table S8: **Full model output of the analysis including low- and high-performing participants in the E2 test.** The reference level for the predictor “condition” was control and for the predictor “performer” was low.

Effect	Group	Term	b	SE	z	*p*-value
fixed	–	(Intercept)	-0.67	0.21	-3.21	.001
fixed	–	conditionHIIT	0.41	0.18	2.27	.023
fixed	–	conditionMICT	0.19	0.21	0.89	.373
fixed	–	performerhigh	1.98	0.29	6.91	< .001
fixed	–	conditionHIIT:performerhigh	-0.35	0.27	-1.31	.192
fixed	–	conditionMICT:performerhigh	-0.36	0.30	-1.21	.225
random	item	sd (Intercept)	1.00	–	–	–
random	item	cor (Intercept).performerhigh	0.53	–	–	–
random	item	sd performerhigh	0.44	–	–	–
random	subject	sd (Intercept)	0.83	–	–	–
random	subject	cor (Intercept).conditionHIIT	-0.31	–	–	–
random	subject	cor (Intercept).conditionMICT	-0.27	–	–	–
random	subject	sd conditionHIIT	0.70	–	–	–
random	subject	cor conditionHIIT.conditionMICT	0.59	–	–	–
random	subject	sd conditionMICT	0.78	–	–	–

Supplementary Table S9: **Pairwise comparisons results from the estimated marginal means.** Pairwise comparisons for the analysis of the effect of exercise on low- and high-performing participants on E2 data.

**Contrast**	**EMM**	**SE**	***z***	***p*-value**
**HIIT – Control** (low)	0.41	0.18	2.27	0.060
**MICT – Control** (low)	0.19	0.21	0.89	0.646
**MICT – HIIT** (low)	-0.22	0.19	-1.19	0.457
**HIIT – Control** (high)	0.06	0.20	0.31	0.949
**MICT – Control** (high)	-0.17	0.21	-0.82	0.688
**MICT – HIIT** (high)	-0.23	0.20	-1.17	0.471

Supplementary analysis 5

The results of the PVT analysis can be found in the Supplementary Table S10. The best-fitting model was: *reaction speed ~ condition + test + (1 + condition + test* | *subject)*. The reference level for the predictor "condition" was control and for the predictor "test" was E1.

Supplementary Table S10: **Model output for the PVT analysis.** Fixed and random effects derived from the analysis of reaction speed measured in the Psychomotor Vigilance Task (PVT).

**Effect**	**Group**	**Term**	**b**	**SE**	**t**	**df**	***p*-value**
fixed	–	(Intercept)	3.11	0.05	60.03	39.10	< .001
fixed	–	conditionHIIT	0.03	0.03	0.91	36.56	.369
fixed	–	conditionMICT	0.03	0.03	1.19	33.97	.242
fixed	–	Test24h	-0.01	0.02	-0.64	46.94	.526
fixed	–	Test2nd	-0.07	0.02	-3.93	73.48	< .001
random	subject	sd (Intercept)	0.31	–	–	–	–
random	subject	cor (Intercept).conditionHIIT	-0.34	–	–	–	–
random	subject	cor (Intercept).conditionMICT	-0.15	–	–	–	–
random	subject	cor (Intercept).Test24h	0.10	–	–	–	–
random	subject	cor (Intercept).Test2nd	0.19	–	–	–	–
random	subject	sd conditionHIIT	0.18	–	–	–	–
random	subject	cor conditionHIIT.conditionMICT	0.35	–	–	–	–
random	subject	cor conditionHIIT.Test24h	-0.29	–	–	–	–
random	subject	cor conditionHIIT.Test2nd	0.22	–	–	–	–
random	subject	sd conditionMICT	0.13	–	–	–	–
random	subject	cor conditionMICT.Test24h	0.09	–	–	–	–
random	subject	cor conditionMICT.Test2nd	0.87	–	–	–	–
random	subject	sd Test24h	0.06	–	–	–	–
random	subject	cor Test24h.Test2nd	-0.24	–	–	–	–
random	subject	sd Test2nd	0.05	–	–	–	–
random	Residual	sd Observation	0.12	–	–	–	–

Supplementary analysis 6

To quantify the internal training load experienced during each intervention, we calculated the training impulse (TRIMP) for each participant using Banister's method (Banister, 1991). The equation used is provided as *Equation 1*. The best-fitting model was: *TRIMP ~ condition + test + (1* **|** *subject)*. The reference level for the predictor "condition" was control. The TRIMP results can be found in the Supplementary Table S11.

Equation 1.

$$TRIMP = t\,.\,k\,.\,x \begin{cases} \dfrac{for\ men\colon k = 0.64\,.\,e^{1.92x}}{for\ women\colon k = 0.64\,.\,e^{1.92x}} \end{cases}$$

Where *t* = duration of training (minute); *k* = sex-dependent intensity factor and *x* = fractional elevation of the maximum HR range (ΔHR_{ratio}), presented in *Equation 2*.

Equation 2.

$$x = \Delta HR_{ratio} = \frac{HR_{exercise} - HR_{rest}}{HR_{max} - HR_{rest}}$$

Supplementary Table 11: The mean (M) and standard deviation (SD) values for the TRIMP are shown for each condition.

	**HIIT**	**MICT**
**TRIMP (M ± SD)**	(28.9 ± 6.0)	(36.8 ± 11.0)
